# Influence of COVID-19 on treatment adherence and psychological well-being in a sample of hypertensive patients: a cross-sectional study

**DOI:** 10.1186/s12888-022-04473-2

**Published:** 2023-02-23

**Authors:** Soledad Gómez-Escalonilla Lorenzo, Isabel Martínez, Blanca Notario Pacheco

**Affiliations:** 1 University of Castilla-La Mancha, Cuenca, Spain; 2 Health Service of Castilla-La Mancha, Toledo, Spain; 3 Department of Psychology, University of Castilla-La Mancha, Cuenca, Spain; 4 Universidad of Castilla-La Mancha, Faculty of Nursing, Cuenca, Spain; 5 Universidad of Castilla-La Mancha, Social and Health Research Center, Cuenca, Spain

**Keywords:** Assessment before the COVID-19 lockdown, Assessment after the COVID-19 lockdown, Hypertension, Treatment Adherence, Psychological Resilience, Self-esteem, Health related Quality of Life

## Abstract

**Objective:**

The COVID-19 pandemic has caused a global health crisis. This situation has affected the general population, especially the most vulnerable populations such as individuals with cardiovascular diseases. The main objective of this study was to analyse adherence to treatment and psychological well-being in hypertensive patients before and after the COVID-19 lockdown in Spain.

**Methods:**

A cross-sectional study was performed in a Basic Health Area of Toledo, Spain. Adherence and psychological well-being (resilience, self-esteem, and health-related quality of life [HRQoL]) were measured in hypertensive patients, a group of patients before the COVID-19 lockdown and, in another group after the COVID-19 lockdown using a heteroadministered and anonymous questionnaire.

A factorial multivariate analysis of variance (MANOVA) was applied for the outcome variables using pre- and post-COVID-19 lockdown assessment, gender, and age (< 65 years-old vs. ≥ 65 years-old) as independent variables. Univariate F follow-up tests were conducted within the multivariate significant overall differences.

**Results:**

The sample of the present study included 331 hypertensive patients. The mean age was 67.68 years (SD = 10.94). Women comprise 53.5% of the sample and men account for the remaining 46.5%. A total of 144 questionnaires were collected before the COVID-19 pandemic and 187 questionnaires were collected after the onset of the pandemic and once the lockdown was over. MANOVA showed significant main effects for pandemic lockdown (*F* = 13.383, *p* < 0.001,) age group (*F* = 3.74, *p* = 0.003) and gender (*F* = 8.85, *p* < 0.001).

Therapeutic adherence decreased after the lockdown (*F* = 15.393, *p* < 0.001). However, scores on resilience (*F* = 17.771, *p* < 0.001), self-esteem (*F* = 4.789, *p* = 0.029), and physical component of HRQoL (*F* = 13.448, *p* < 0.001) increased after the lockdown.

Regarding age, the univariate test showed a significant effect for the physical component of HRQoL, with scores decreasing in those aged ≥ 65 years (*F* = 9.375, *p* = 0.002). Regarding gender, women scored lower on resilience (*F* = 20.280 *p* < 0.001), self-esteem (*F* = 18.716, *p* < 0.001), the physical component of HRQoL (*F* = 5.722, *p* = 0.017), and the mental component of HRQoL (*F* = 28.912, *p* < 0.001).

**Conclusions:**

The COVID-19 pandemic had a negative effect on treatment adherence of hypertensive patients in Spain. However, variables related to psychological well-being have increased in these patients, which may serve as a protective factor against pandemic stress.

## Introduction

In 2020, the coronavirus disease 2019 (COVID-19) pandemic was a major global health crisis. Many individuals have been affected by the virus itself, which has caused a great deal of death and suffering, as well as by social and economic hardships [1, 2]. To prevent the spread of the virus, various measures were taken that affected people's daily lives, causing considerable psychological stress. These measures triggered widespread anxiety, depression, anguish, insomnia, and fear in the general population [3–5]. In Spain, during the lockdown, the uncertainty of the situation experienced by people with chronic illness caused, negative feelings increase by 40 percentage points [6]. However, some research shows that there is no substantial change in psychological and spiritual outcomes over time during the COVID-19 pandemic, rather, an increase in psychological and spiritual resilience was observed [7–11]. In addition, studies with survivors of other disasters (other pandemics, natural disasters, terrorist attacks, etc.) show levels of positive psychological functioning [12–14], experiencing a sense of control, self-esteem and belonging, by providing emotional support to others [14].

In this study, psychological well-being (resilience, self-esteem, and health-related quality of life (HRQoL) was analysed along with adherence to treatment in hypertensive patients before and after the COVID-19 pandemic lockdown in Spain. Resilience has been described as the ability to adapt to changes in life circumstances and provide flexible responses to daily stress [15, 16]. Moreover, it has been demonstrated that resilience acts as a protective factor against mental health problems [17, 18] and could play a key role in the experience and management of chronic comorbidities [19].

Self-esteem is an individual’s set of thoughts and feelings about him/herself and the degree of self-approval or refusal [20]. Self-esteem, as the person’s perception of him/herself, is formed through experiences with the environment [21], and it is especially influenced by significant others and environmental reinforcements [22, 23]. As the core of the individual, self-esteem has been considered essential in understanding emotional, cognitive, and behaviour functioning [24, 25]. The person’s view of himself or herself can directly influence the behaviour associated with the disease. It has been observed that higher levels of self-esteem are associated with patient motivation to improve their self-care behaviour, contributing to symptom control and therefore to an improvement in patient attitude [26].

Health-related quality of life (HRQoL) is the impact of a disease or medical condition on physical, psychological, and social dimensions of health that are influenced by one’s own experiences, beliefs, expectations, and perceptions [7, 27, 28]. HRQoL is widely assessed in patients with chronic disease, especially in hypertensive patients, and it has been observed that hypertensive patients tend to have a lower HRQoL than normotensive individuals [29, 30]. This situation may have been aggravated by the strategies implemented to contain the COVID-19 pandemic. 

Simultaneously, the crisis in cardiovascular disease (CVD) and its main risk factor, uncontrolled hypertension, has continued, reaching pandemic proportions. Thus, after decades of reductions in premature CVD mortality, a significant slowdown in this process has been documented in high-income countries [2, 31]. Some particularly vulnerable populations are identified, such as people aged 65 years and over, who account for 18.5% of the population in the European Union, as this age group has multiple morbidities, with multiple concurrent CVDs [3, 32]. Hypertension is one of the most common diseases in the world, and a major cause of death and disability [1, 3]. Hypertension is associated with symptoms of depression and anxiety [33]. During the COVID-19 pandemic, several risk factors for increased blood pressure (BP) were observed: social distancing, economic changes, changes in sleep patterns and lifestyle changes such as reduced physical activity [34, 35].

On the other hand, changes made to the health services to cope with the COVID-19 pandemic have isolated many chronically ill patients from routine care [34]. In Spain, 30.7% of patients with chronic diseases experienced different problems in obtaining their medication during the lockdown. In addition, approximately 25% of respondents reported that they occasionally forgot to take their medication, and this phenomenon was more frequently noted in women [6]. Another problem for hypertensive patients related to a controversial issue that arose in March 2020 regarding the possible adverse effects of angiotensin-converting enzyme inhibitors (ACE inhibitors) or angiotensin II receptor blockers (ARBs), which prompted the European Society of Cardiology to issue a statement recommending physicians and patients not to discontinue antihypertensive treatment [32, 34, 36].

There is a paucity of research in Spain on how the COVID-19 pandemic has affected adherence to treatment in hypertensive patients [6, 35]. Moreover, there are mixed results regarding the impact on the mental health of the population during this crisis [3–11]. The objective of this study was to analyse adherence to treatment and psychological well-being (resilience, self-esteem, and health-related quality of life [HRQoL]) in hypertensive patients before and after COVID-19 pandemic lockdown in Spain, based on the gender and age of the participants.

## Methods

### Study design

This cross-sectional study was performed from March 2019 to March 2021 in a Basic Health Area of Toledo, Spain. Adherence and psychological well-being (resilience, self-esteem, and HRQoL) were measured in hypertensive patients, a group of patients before the COVID-19 pandemic lockdown and, another group of patients after the COVID-19 lockdown.

### Participants

The study population comprised hypertensive patients from a basic health area in central Spain. The inclusion criteria were as follows: a) over 18 years of age; b) registered with a health card in health area; c) diagnosed with hypertension in the period of data collection; and d) normal cognitive capacities to provide informed consent. On the other hand, the exclusion criteria were as follows: a) refusal to participate in the study; and b) inability to communicate in Spanish.

### Sampling and sample size

Using convenience sampling, participants were recruited from the different nursing offices in this health area. A priori power analysis determined that 314 participants yielded an unfavour small effect size (f = 0.5) with a power of 0.95 (α = 0.05, 1—β = 0.95) in the F-test. A total of 331 hypertensive patients completed the different scales after providing informed consent to participate in this study. A total of 144 questionnaires were collected before the COVID-19 pandemic, and 187 questionnaires were collected after the onset of the pandemic and once the lockdown was over. The participation rate before COVID-19 lockdown was 98% and after lockdown 97%.

### Ethical considerations

The present study was approved by the Clinical Research Ethics Committee of the Area of Health from Toledo, Spain (Reg. 318) and all research was performed in accordance with the regulations of this committee. Study participants were all informed about the objectives of the study, and written informed consent was obtained prior to individual participation.

### Data collection

A structured questionnaire comprising 51 items was used. Questions were divided into 6 domains: demographics, medical variables, therapeutic adherence was assessed with the Spanish version of Martin-Bayarre-Grau questionnaire, Resilience was evaluated with the Spanish version of Health and Quality of Life Outcomes (10-item CD-RISC), Self-esteem was assessed with the 10-item Spanish version of the Rosenberg self-esteem scale, and health-related quality of life was evaluated with the SF-12 Health Questionnaire.

All heteroadministered questionnaires were completed anonymously following the clinical guidelines of the Clinical Research Ethics Committee of the Area of Health from Toledo, Spain. All participants (100%) completed all the items on the questionnaire.

### Demographics

Demographic variables included age, gender, marital status, and education level.

### Medical variables

Medical variables included years of hypertension, comorbidities, and whether participants had past COVID-19 infections.

Therapeutic adherence was evaluated with the Spanish version of Martin-Bayarre-Grau therapeutic adherence questionnaire [37]. The scale includes 12 items (e.g., “Take the medicines at the prescribed established timetable”) that assess levels of adherence to treatment in individuals with hypertension. The Likert response scale has 5 points ranging from 0 “never” to 4 “always”. The total score ranges from 0 to 48. Complete adherence was noted for participants who obtain 48 to 38 points, partial adherence was noted for 37 to 18 points and non-adherence was noted for those who score 17 to 0 points. This questionnaire was validated in Spanish [37] and has been used in hypertensive populations [38]. The alpha value was 0.889.

Resilience was evaluated with the Spanish version of the Health and Quality of Life Outcomes (10-item CD-RISC) [39]. The scale includes 10 items (e.g., “Able to adapt to change”) that assess resilience, which has been defined as a protective factor against mental problems and as a dynamic process of adaptation to changes in life circumstances. The Health and Quality of Life Outcomes survey is a self-administered questionnaire that are answered using Likert type additive scale with five response options (0 = never; 4 = almost always). The questionnaire contains a single dimension that appears in the original version [40]. The final score on the questionnaire was the sum of the responses obtained on each item (range 0–40), and higher scores indicate higher levels of resilience. The alpha value was 0.77.

Self-esteem was evaluated using the 10 item Spanish version of the Rosenberg self-esteem scale [41], which was originally developed by Rosenberg (1965) for the assessment of self-esteem in adolescents (e.g., “On the whole, I am satisfied with myself”). Responses are provided using a Likert response scale of 4 points (ranging from 1 = strongly agree to 4 = strongly disagree). The scale has an equal number of positively and negatively worded items. The total score ranges between 10 and 40. The alpha value was 0.83.

Health-related quality of life was evaluated with the SF-12 Health Questionnaire originally developed by McHorney et al. [42] and adapted in Spain by Alonso et al. [43] The SF-12 Health Questionnaire is a reduced version of the SF-36 Health Questionnaire [44]. The questionnaire comprises twelve items that assess the degree of well-being and functional capacity of people over 14 years of age. The SF-12 includes physical and mental component summary scores. The alpha value was 0.76.

### Statistical analysis

A factorial multivariate analysis of variance (MANOVA) was applied for the following outcome variables: adherence treatment, resilience, self-esteem and HRQL (physical and mental dimension), pre- and post- COVID-19 lockdown assessment (before the COVID-19 pandemic vs. after the lockdown of the COVID-19 pandemic), sex (men vs. women), and age (< 65 years-old vs. ≥ 65 years-old) served as independent variables. Univariate F follow-up tests were performed, and the multivariate significant overall differences were identified.

## Results

### Survey respondents

A total of 331 participants responded to the questionnaire. The participants ranged in age from 35 to 90 years old. The mean age was 67.68 (standard deviation (SD) = 10.94). Women comprised 53.5% of the sample, and men accounted for the remaining 46.5%.

Table 1 shows the demographic and medical characteristic of the participants in the two study groups (before and after of the COVID-19 pandemic lockdown).

**Table 1:** Demographic and medical characteristics

Characteristics	Before the pandemic lockdown	After the pandemic lockdown	Total
	*n* = 144	*n* = 187	*n* = 331
Age			
Mean ± SD (Range)	68.24 ± 11.57 (35–90)	67.25 ± 10.44 (37–88)	67.68 ± 10.94 (35–90)
Gender n(%)			
Male	70 (48.6)	84 (44.9)	154 (46.5)
Female	74 (51.4)	103 (55.1)	177 (53.5)
Marital status n(%)			
Married	117 (81.3)	142 (75.9)	259 (78.2)
Unmarried	27 (18.7)	45 (24.1)	72 (21.8)
Education level n(%)			
Tertiary	7 (4.9)	17 (9.1)	24 (7.3)
Secondary	20 (13.9)	30 (16)	50 (15.1)
Primary	80 (55.6)	105 (56.1)	185 (55.9)
Without studies	37 (25.7)	35 (18.7)	72 (21.7)
Years of Hypertension			
Mean ± SD (Range)	12.72 ± 9.96 (0.08–50)	10.99 ± 7.71 (0.75–40)	11.74 ± 8.79 (0.08–50)
Comorbidities n(%)			
Hyperlipemia	88 (61.11)	112 (59.89)	200 (60.42)
Obesity	60 (41.66)	85 (45.45)	145 (43.8)
Diabetes	50 (34.72)	51 (27.27)	101 (30.51)
Arthrosis	27 (18.75)	64 (34.22)	91 (27.49)
Depression	19 (13.19)	14 (7.48)	33 (9.96)
Tobacco use	12 (8.33)	15 (8.02)	27 (8.15)
Covid-19 n(%)			
Yes		16 (8.6)	16 (4.8)
No		171 (91.4)	315 (95.2)

*SD* Standard deviation

### Multivariate analysis: COVID-19 pandemic lockdown, age, and sex effects

MANOVA showed significant main effects for pandemic lockdown, (ʌ = 0.827, *F* (5, 319) = 13.383, *p* < 0.001), age group (ʌ = 0.945, *F* (5, 319) = 3.74, *p* = 0.003) and gender (ʌ = 0.878, *F* (5, 319) = 8.85, *p* < 0.001) (see Table 2). No interaction among the three independent variables was observed. Descriptive values of the outcome variables are presented in Table 3.

**Table 2:** MANOVA: COVID-19 pandemic lockdown, age, and gender effects

Source of variation	Λ	*F*	*gl*_between_	*gl*_error_	*P*
Intersection	0.008	8095.75	5	319.00	< 0.001
(A) Covid-19 Lockdown	0.827	13.38	5	319.00	< 0.001
(B) Gender	0.878	8.85	5	319.00	< 0.001
(C)Age	0.945	3.74	5	319.00	0.003
A x B	0.974	1.69	5	319.00	0.135
A x C	0.979	1.37	5	319.00	0.235
B x C	0.991	0.577	5	319.00	0.718
A x B x C	0.989	0.703	5	319.00	0.621

**Table 3:** Outcome variables: Means, standard deviations (SD), maximum and minimum values

Outcomes variables	Means	SD	Minimum	Maximum
Adherence Treatment	34.73	3.866	21	48
Resilience	31.57	7.092	7	40
Self-esteem	36.94	4.585	10	40
Physical component	42.36	9.093	14.59	57.43
Mental component	41.9	8.986	8.8	59.05

### Effects of the COVID-19 pandemic lockdown on therapeutic adherence and psychological well-being

The univariate test showed a significant effect for therapeutic adherence (*F* (1, 323) = 15.393, *p* < 0.001), with scores decreasing after the pandemic lockdown. However, scores for resilience (*F* (1, 323) = 17.771, *p* < 0.001); self-esteem, (*F* (1, 323) = 4.789, *p* = 0.029) and the physical component of HRQoL (*F* = (1, 323) 13.448, *p* < 0.001) increased after the pandemic lockdown. Scores on the mental component of HRQoL did not significantly differ among the groups (see Table 4).

**Table 4:** Means, standard deviations (SD), *F* Values, and Probabilities of a Type I Error for the effect of pandemic lockdown on the outcome variables

COVID-19 pandemic lockdown				
Source of variation	Before pandemic	After pandemic	*F*	*p*
Therapeutic adherence	35.63 (4.2)	34.50 (3.09)	15.393	< 0.001
Resilience	29.92 (7.72)	32.84 (6.29)	17.771	< 0.001
Self-esteem	36.46 (4.61)	37.32 (4.53)	4.789	0.029
Physical component HRQoL	40.34 (10.01)	43.92 (7.99)	13.448	< 0.001
Mental component HRQoL	42.72 (9.05)	41.26 (8.9)	2.102	0.148

*HRQoL* Health-related quality of life

### Gender and age effects

Regarding age, the univariate test showed a significant effect for the physical component of HRQoL, with scores decreasing in those aged ≥ 65 years, (*F* (1, 323) = 9.375, *p* = 0.002) (see Table 5).

**Table 5:** Means, standard deviations (SD), *F* Values and Probabilities of a Type I Error for the effect of age in the outcome variables

Age groups				
Source of variation	< 65	≥ 65	*F*	*p*
Therapeutic adherence	34.23 (3.892)	35.05 (3.825)	2.631	0.106
Resilience	31.46 (7.330)	31.64 (6.953)	0.756	0.385
Self-esteem	36.60 (5.584)	37.16 (3.813)	2.498	0.115
Physical component HRQoL	44.49 (8.961)	41.01 (8.937)	9.375	0.002
Mental component HRQoL	41.04 (9.377)	42.44 (8.706)	2.280	0.132

*HRQoL* Health-related quality of life

Regarding gender, the univariate test showed a significant effect for resilience (*F* (1, 323) = 20.280, *p* < 0.001), self-esteem (*F* (1, 323) = 18.716, *p* < 0.001), the physical component of HRQoL (*F* (1, 323) = 5.722, *p* = 0.017), and the mental component of HRQoL (*F* (1, 323) = 28.912, *p* < 0.001), with scores decreasing in women (see Table 6).

**Table 6:** Means, standard deviations (SD), *F* Values and Probabilities of a Type I Error for the effect of gender in the outcome variables

Gender				
Source of variation	Men	Women	*F*	*P*
Therapeutic adherence	34.88 (3.995)	34.60 (3.757)	0.385	0.535
Resilience	33.21 (5.785)	30.14 (7.797)	20.280	< 0.001
Self-esteem	38.03 (2.882)	35.99 (5.501)	18.716	< 0.001
Physical component HRQoL	43.84 (9.015)	41.08 (8.991)	5.722	0.017
Mental component HRQoL	44.81 (7.741)	39.36 (9.241)	28.912	< 0.001

*HRQoL* Health-related quality of life

## Discussion

This study has advanced scientific knowledge on the impact of the COVID-19 pandemic lockdown on hypertensive patients, in terms of adherence to treatment and psychological well-being. Prospective, longitudinal research on these effects are lacking.

Among the hypertensive patients participating in this study, a decrease in therapeutic adherence was observed after the COVID-19 pandemic lockdown. Regarding psychological variables, resilience, self-esteem, and the physical component of HRQoL increase, whereas the mental component of HRQoL decreased slightly. In those aged ≥ 65 years, the physical component of HRQoL significantly decreased, whereas adherence to treatment, resilience, self-esteem, and the mental component of HRQoL increased. In women, all the variables studied decreased. The reductions in resilience, self-esteem, and the physical and the mental component of HRQoL were significant.

The main strengths of this study were that the results confirm the adverse impact of the pandemic on chronic patients [2, 6, 34, 35, 45], namely, hypertensive patients, and a negative effect on adherence to treatment was observed. On the other hand, an improvement was found in variables related to psychological well-being, which is consistent with some research conducted after the COVID-19 pandemic lockdown [8–11]. Vulnerable groups were identified as individuals aged ≥ 65 years of age and women [1, 3, 6, 35, 45].

### Adherence to treatment in hypertensive patients

Adherence to treatment in this sample of hypertensive patients decreased after the COVID-19 lockdown in Spain. These results are consistent with other research describing lower adherence during the COVID-19 pandemic in individuals with chronic diseases [6, 46–48], due to restrictions that occurred during the pandemic, such as difficulties in accessing face-to-face medical visits, problems in obtaining medication and poor medication adherence. In a recent study in the United States [48], 51% of people with chronic diseases reported medication-related problems, with 19.6% reporting problems obtaining medication and 31.7% reporting forgetting to take or not taking their medication.

In addition, the lockdown and fear of COVID-19 have resulted in a lack of exercise for these patients, which is an important part of their treatment [6, 34, 35]. Changes in adherence to the recommended diet for hypertensive patients were also noted. For example, these patients reported an increase in sodium intake, associated with an increase in processed products, and patients reported eating more during confinement, leading to an increase in their body mass index (BMI) [2, 35]. Clinical differences in adherence were noted based on age group and gender, with greater adherence observed in those over 65 years of age, which is consistent with other research [49, 50]. Regarding gender, lower adherence was noted in women, which is also observed in other studies of individuals with chronic diseases [48, 51, 52].

### Psychological well-being in hypertensive patients

Contrary to other research [1, 3, 6, 35, 36], the effect of the COVID-19 pandemic lockdown on the psychological well-being of these patients includes increases in resilience, self-esteem, and the physical component of HRQoL. It should be noted that most of these studies measure anxiety and depressive symptoms as indicators of psychological well-being [1, 3–5]. On the other hand, the results are consistent with previous prospective longitudinal studies on natural disasters [7] and the COVID-19 pandemic [8–11], these studies have reported relatively stable trajectories of psychological outcomes following disasters. In a prospective longitudinal study of adults with chronic disease during the COVID-19 pandemic, an increase in psychological and spiritual resilience was also found [7]. A recent study in the Netherlands reported no changes in the HRQoL of individuals with other chronic diseases after the first lockdown due to the COVID-19 pandemic [53].

In the group aged ≥ 65 years, a decrease in the quality of life is observed, which is similar to that noted in other research [45, 54], because older people are at a significantly higher risk of serious disease. The physical dimension of HRQoL has decreased after the pandemic [54], and is, possibly related to the decrease in physical activity that occurred during lockdown [3, 6, 45]. Slight increases in the mental dimension of HRQoL, resilience and self-esteem, were noted, which is consistent with previous studies in which older adults reported lower overall levels of depression, anxiety, and stress compared with younger age groups [3, 55].

Considering the gender perspective, women obtained lower scores for all variables related to psychological well-being, which is consistent with other research. These findings identify women as one of the vulnerable groups in the context of the psychological and emotional impact of the COVID-19 pandemic [1, 3, 6, 35].

### Limitations of the study

This research presents some limitations. The present study employed a cross-sectional design and thus could not capture changes in therapeutic adherence or in psychological well-being variables over the course of the COVID-19 outbreak. The use of convenience sampling and its descriptive nature may not allow the generalization of results.

Despite these limitations, the study clearly shows the negative effect of the COVID-19 pandemic lockdown on adherence in this group of patients, and a positive effect on psychological well-being after lockdown.

## Conclusions

The COVID-19 pandemic has had a negative effect on the treatment adherence of hypertensive patients in Spain. This finding may have important implications for the health of these patients, as it may increase the risk of complications related to poor control of their chronic disease. However, the fact that psychological well-being has increased in these patients may represent a protective factor against the stress triggered by the pandemic.

In individuals over 65 years of age, an impairment of the physical dimension of HRQL is observed that can lead to further physical deterioration. This aspect is very important in hypertensive patients given that physical exercise is one of the components of antihypertensive treatment.

Faced with this reality, health systems need to be alert and design strategies to improve the care provided to patients at high cardiovascular risk to reduce the negative impact of the health crisis due to the COVID-19 pandemic.

## Data Availability

All data collected and analysed during the current study are available from the corresponding author on reasonable request.

## Abbreviations

COVID-19: Coronavirus Disease 2019
CVD: Cardiovascular disease
BP: Blood pressure
ACE inhibitors: Angiotensin-converting enzyme inhibitors
ARBs: Angiotensin II receptor blockers
HRQL, HRQoL: Health-related quality of life
SD: Standard deviation
MANOVA: Multivariate analysis of variance
BMI: Body mass index
